# Fünfzehn-Jahres Ergebnisse nach Monitoring, Operation oder Strahlentherapie aufgrund eines Prostatakarzinoms

**DOI:** 10.1007/s00066-024-02210-0

**Published:** 2024-01-30

**Authors:** Simon K. B. Spohn, Anca-Ligia Grosu

**Affiliations:** Klinik für Strahlenheilkunde, Universitätsklinikum Freiburg, Robert-Koch-Straße 3, 79106 Freiburg, Deutschland

## Hintergrund

Die ProtecT Studie hat durch PSA-Testung 2664 Patienten mit lokalisiertem Prostatakarzinom (PCa) diagnostiziert und 1643 von Ihnen zwischen aktivem Monitoring (AM) (*n* = 545), Prostatektomie (RPE) (*n* = 553) und Strahlentherapie (RT) (*n* = 545) randomisiert. Die aktuelle Studie berichtet über die Ergebnisse nach einem medianen Follow-Up von 15 Jahren. Neben onkologischen Endpunkten, wie prostatakrebs-spezifischem Überleben, Metastasen-freiem-Überleben, progressionsfreiem-Überleben und dem Beginn einer Langzeit-Androgendeprivationstherapie (ADT) erfolgte eine zusätzliche Analyse der Risikoklassifizierung.

## Methoden

Zwischen 1999 und 2009 wurden 82.429 Patienten zwischen 50 und 69 Jahren im Vereinigten Königreich in die ProtecT Studie eingeschlossen. Bei 2664 von Ihnen wurde ein lokalisiertes PCa diagnostiziert. Bei Patienten in der AM Gruppe führte eine Erhöhung des PSA-Werts von mind. 50% innerhalb von 12 Monaten oder Unbehagen von Seiten des Patienten oder Ärzt*innen zu einer erneuten Evaluation des Therapieregimes. Im Falle einer R1 Resektion, extrakapsulären Durchbruchs oder PSA-Werten > 0,2 ng/ml nach RPE erfolgte das Angebot einer adjuvanten RT. Die RT wurde nach neoadjuvanter ADT von 3–6 Monaten mittels 3D-Bestrahlungstechnik und 74 Gy in 37 Fraktionen appliziert.

Der primäre Endpunkt der Studie war Tod aufgrund des PCa. Sekundäre Endpunkte umfassten Tod jeder Ursache, das Vorhandensein von Metastasen (bildgebend oder PSA > 100 ng/ml), klinischer Progress, klinischer V. a. T3 oder T4 Erkrankung, Initiierung einer Langzeit ADT, Urethra-Obstruktion, rektale Fisteln und die Anlage von Kathetern aufgrund von Tumorwachstum.

Die Statistik wurde mittels Cox Regression auf Basis einer Intention-to-treat Analyse durchgeführt. Innerhalb der 488 Patienten, die mittels RPE behandelt wurden erfuhren 138 (28,5%) ein Upgrading zu einem pT3 oder pT4 Stadium und 155 Patienten (32,0%) eine höheres pathologisches Grading. 245 (50,5%) der Patienten hatten einen Gleason-Score von 3 + 4 = 7a oder höher.

## Ergebnisse

Nach medianem Follow-Up von 15 Jahren waren die klinischen Daten für 1610 von 1643 Patienten vollständig vorliegend. Nach der D’Acimo Klassifizierung hatten 369 Patienten (24,1%) ein intermediäres und 147 (9,6%) ein hohes Risikoprofil. Nach CAPRA Kriterien hatten 428 Patienten (26,4%) ein intermediäres und 40 (2,5%) ein hohes Risikoprofil.

45 Patienten (2,7%) starben aufgrund des PCa, davon 17 (3,1) in der AM-Gruppe, 12 (2,2%) in der RPE-Gruppe und 16 (2,9%) in der RT-Gruppe. Der Unterschied war statistisch nicht signifikant (*p* = 0,53). 7 der 16 Todesfälle in der RT-Gruppe traten nach 15 Jahren FU auf. 104 Männer (6,3%) entwickelten Metastasen, wovon 51 (9,4%) in der AM-Gruppe, 26 (4,7%) in der RPE-Gruppe und 27 (5,0%) in der RT-Gruppe waren. 151 Männer (9,2%) erhielten eine Langzeit-ADT, wovon 69 (12,7%) in der AM-Gruppe, 40 (7,2%) in der RPE-Gruppe und 42 (7,7%) in der RT-Gruppe waren. 259 Männer (9,2%) erlitten einen lokalen Progress, wovon 141 (25,9%) in der AM-Gruppe, 58 (10,5%) in der RPE-Gruppe und 60 (11,0%) in der RT-Gruppe waren. Während Tode anderer Ursache statistisch nicht signifikant unterschiedlich zwischen den Gruppen war, hatten Patienten die eine RPE oder RT erhielten statistisch signifikant geringere Risiken für die Entstehung von Metastasen (Hazard Ratio = 0,47 bzw. 0,48), die Einleitung einer Langzeit-ADT (Hazard Ratio = 0,54 bzw. 0,54) oder lokalen Progress (Hazard Ratio = 0,36 bzw. 0,35). Insgesamt hatten nach Abschluss des Follow-Up 133 Männer (24,4%) keine aktive Therapie erhalten und waren am Leben.

## Schlussfolgerung der Autoren

Nach einem medianen Follow Up von 15 Jahren waren die Mortalität von PSA-detektierten PCa sehr niedrig, unabhängig davon welches Therapieregime die Patienten erhielten. Aktive Therapien haben zwar zu einer geringeren Rate an Progress geführt aber das Gesamtüberleben nicht beeinflusst und das obwohl das verwendete *active monitoring* Protokoll im Vergleich zu heute empfohlenen *active surveillence *Protokollen weniger intensiv war. Ein Viertel der Patienten im *active-monitoring* Arm waren am Leben und haben keine Therapie erhalten. Zusätzlich zeigen die Ergebnisse, dass aktuelle Risikoklassifizierungen Limitationen aufweisen und ca. 1/3 der Patienten innerhalb der Studie nach aktueller Klassifizierung als intermediäres oder hohes Risikoprofil eingestuft würden. Entsprechende letale Faktoren müssen in zukünftigen Studien identifiziert werden. Risiken und Nutzen sollen bei der Therapieentscheidung vorsichtig abgewogen werden.

## Kommentar

Die Protect-Studie ist eine sehr wichtige und richtungsweisende Studie. Die präsentierten Daten mit einem nun langen Follow-Up von 15 Jahren können Ärzten helfen, den *shared-decision-making*-Prozess zu verbessern und mit den Patienten den Nutzen einer Therapie und mögliche Nebenwirkungen zu diskutieren. *Active Monitoring* bzw. *active surveillance* stellt eine wichtige Therapieoption für Patienten mit niedrigem und ggf. intermediärem Risikoprofil dar und sollte als solche auch diesen Patienten angeboten werden.

Gleichzeitig muss die signifikant reduzierte Rate an Progression und Entstehung von Metastasen durch eine aktive Therapie gegenüber der Überwachung berücksichtig werden. Auch wenn diese lange Zeit (in dieser Studie über einen Zeitraum von immerhin 15 Jahren) nicht letale Konsequenzen haben, sind *Salvage*-Behandlungen mit Morbidität, psychischer Belastung und zusätzlichen Kosten verbunden. Die Bedeutung der psychischen Komponente bei der Therapieentscheidung wird auch durch die Tatsache bekräftigt, dass Patienten von der aktiven Überwachung in eine Therapie häufig nicht aufgrund medizinischer Befunde, sondern persönlichen Gründen, wie Sorgen, übergingen.

Bei der finalen Therapieentscheidung sind insbesondere die unterschiedlichen Nebenwirkungsprofile der Therapien zu berücksichtigen. In einer weiteren Publikation der Gruppe wurde die Lebensqualität nach 12 Jahren evaluiert [1]. Diese Arbeit hat gezeigt, dass Patienten die mittels RT behandelt werden eine besserer Urinkontinenz und Erektion aufweisen als Patienten die mittels RPE und AM (häufig war die gewählte Therapie bei Initiierung einer Behandlung eine RPE) behandelt worden sind. Bei der Interpretation dieser Daten müssen technische Fortschritte in den Therapien, wie die nervenschonende und roboterassistierte Operation sowie die Verwendung von intensitätsmodulierter Strahlentherapie und hypofraktionierten Schemata, berücksichtigt werden. Erste Ergebnisse der PACE-A-Studie, welche Patienten zwischen Roboter-assistierter OP und stereotaktischer Strahlentherapie (SBRT) randomisiert hat, bestätigen aber die beschriebene Tendenz mit deutlichen Vorteilen der SBRT hinsichtlich Urininkontinenz und nur sehr geringen gastrointestinalen Nebenwirkungen [2]. Eine Subgruppenanalyse der ProtecT-Studie zeigte, dass v. a. Patienten > 65 Jahre von einer frühzeitigen Therapie profitierten. Eine fortschrittliche Strahlentherapie könnte also insbesondere für dieses Patientenkollektiv eine optimale Option darstellen.

Schließlich zeigen die Ergebnisse dieser Studie aber erneut, dass es sich beim PCa um eine sehr heterogene Erkrankung handelt und Risikoklassifizierungen auf Basis von klassischen pathologischen und klinischen Parametern ein unzufriedenstellendes Ergebnis erzielen. Auch die hohe Anzahl von klinischem und histopathologischem Upstaging für Patienten nach RPE unterstreicht die Notwendigkeit einer verbesserten Tumorcharakterisierung. *Artifical-intellegence-*unterstütze Analysen des Genoms (genomic classifier) [3], der Bildgebung (Radiomics) oder digitalisierter Histopathologie bieten diesbezüglich vielversprechende Ansätze und könnten in Zukunft das Therapiemanagement maßgeblich beeinflussen um Patienten eine optimierte und personalisierte Therapie zu ermöglichen und sowohl Über- als auch Untertherapie entgegenzuwirken.

## Fazit

- Nach einem medianen Follow-Up von 15 Jahren zeigt die ProtecT Studie keine Mortalitätsunterschiede zwischen Active Monitoring, radikaler Prostatektomie oder definitiver Strahlentherapie. Dies unterstreicht die Bedeutung der *Active Surveillance*, welche insbesondere für low-risk Patienten eine zu empfehlende Therapieoption ist.
- Patienten, die eine aktive Therapie erhielten, erfuhren aber signifikant weniger Metastasen, lokalen Progress oder eine Langzeit-ADT. Diesbezüglich zeigten sich keine statistisch signifikanten Unterschiede zwischen Strahlentherapie und Operation.
- Diese Erkenntnisse können unter Berücksichtigung neuer Therapieverfahren und -Ergebnisse, sowie des Nebenwirkungsprofils der Therapien helfen eine optimale Therapieentscheidung gemeinsam mit den Patienten zu treffen.
- Insbesondere für Patienten > 65 Jahren erzielt die Strahlentherapie gleiche Therapieerfolge wie die Operation bei geringem Risiko für relevante Nebenwirkungen.
- Weitere prognostische und prädiktive Bio‑/Marker sind notwendig um die Risikoklassifizierung zu verbessen und Patienten zu identifizieren, die von einer aktiven Therapie profitieren und Über- und Untertherapie zu vermeiden.

*Simon K.B. Spohn und Anca-Ligia Grosu, Freiburg, D*
